# Evaluation of overweight control applications with cognitive‐behavioral therapy approach: A systematic review

**DOI:** 10.1002/hsr2.1157

**Published:** 2023-03-27

**Authors:** Negin Ebrahimi, Niloofar Mohammadzadeh, Seyed Mohammad Ayyoubzadeh

**Affiliations:** ^1^ Health Information Management Department School of Allied Medical Sciences, Tehran University of Medical Sciences Tehran Iran

**Keywords:** digital health, exercise, health informatics, nutrition, obesity

## Abstract

**Background and Aims:**

Overweight and obesity lead to the development of physical diseases. Cognitive factors play a vital role in controlling one's weight. Currently, cognitive‐behavioral therapy (CBT) interventions are recognized as a subcategory of lifestyle modification programs that can be implemented to control weight and modify eating patterns as well as physical activity. Nowadays, smartphone‐based applications are utilized to implement behavioral interventions. The main purpose of this study is to evaluate the quality of CBT‐based smartphone applications available on *Google Play* and the *App Store* in the field of overweight control.

**Methods:**

Smartphone‐based utility applications available on *Google Play* and *App Store* were identified in March 2021. Weight control smartphone applications were obtained based on inclusion and exclusion criteria. The app name, platform, version, number of downloads, password protection, affiliations, and features of retrieved apps were tabulated. The Mobile Application Rating Scale was utilized to evaluate the quality of the identified apps.

**Results:**

Seventeen CBT‐based weight control smartphone apps were retrieved. The average engagement, functionality, aesthetics, and information quality scores were 3.65, 3.92, 3.80, and 3.91, respectively. Also, the average score in an aspect containing the usefulness of the app, frequency of using the application, cost, and user satisfaction was 3.5.

**Conclusion:**

Future applications related to this field can be improved by providing a personalization program according to the needs of users and the possibility of online chatting with the therapist. Further improvements can be achieved by improving the areas of engagement, aesthetics, and subjective quality as well as having appropriate privacy policies.

## 1 INTRODUCTION

Being overweight is one of the most significant factors that lead to developing physical diseases such as cardiovascular disease, diabetes, joint damage, lumbago, certain types of cancer, hypertension, fatty liver, and an increased mortality rate.^1^, ^2^ The World Health Organization has classified the prevalence of being overweight as a global epidemic due to the rapidly rising rate of overweight and obesity throughout the globe.^3^ Cognitive factors play a key role in controlling one's weight. Altering these maladaptive cognitions using implementing control of overeating and satiety is highly effective in the success achieved through dieting.^4^ Currently, cognitive‐behavioral therapy (CBT) interventions are recognized as a subcategory of lifestyle modification programs that can be implemented to control weight and modify eating patterns as well as physical activity.^5^ This approach ultimately assists people in altering their eating habits, *not* in the short term but throughout their entire lifetime, by changing their manner of thinking.^6^

Nowadays, smartphone‐based applications are utilized to implement behavioral interventions.^7^ The use of such applications to help control weight is on the rise. However, the quality and quantity of the effectiveness as well as efficiency of these applications have not been entirely determined.^8^ The results of a systematic review by Antoun et al. indicated that mobile‐based interventions aimed at losing weight were more effective on target populations than the control groups by providing a suitable diet, physical activity program, and behavioral indicators that determine weight management.^9^ However, based on a review study, none of these applications have addressed behavioral aspects and evaluated the dysfunctional thoughts that prevent accomplishing the permanent weight loss objective.^10^ Meanwhile, studies have indicated that smartphone devices may be used to boost self‐efficacy by activating specific behavior and altering dysfunctional thoughts in other fields of health.

Companies such as *Apple* and *Google* provide users with numerous health applications through the *App Store* and *Google Play* platforms, respectively. However, only a few of these apps have been approved by the *Food and Drug Administration*.^11^ Furthermore, there are currently no regulations for developing smartphone‐based health applications. Thus, evaluating health apps is of the utmost importance.

Numerous tools are available for evaluating applications, including the *QUIZ tools*, the *Nielsen*, *Norman*, and *Shneiderman* models, as well as the *Mobile Application Rating Scale* (MARS). One of the tools utilized to evaluate smartphone applications, particularly in the field of health applications, is the MARS scale.^12^ This scale consists of 23 items that evaluate the quality of smartphone applications in four qualitative aspects (engagement, functionality, aesthetics, and information quality) and one subjective aspect.^13^ In addition to evaluating applications from a quantitative perspective, this scale also qualitatively evaluates them. This scale has been put into use to evaluate smartphone apps in the field of diabetes,^14^ pregnancy,^15^ epilepsy,^16^ sleep self‐care control,^17^ health behavior change,^18^ COVID‐19 management,^19^ and food allergies.^20^

The main purpose of this study is to evaluate the quality of smartphone applications available on the *Google Play* and *App Store* in the field of overweight control based on CBT by applying the MARS scale.

## 2 MATERIALS AND METHODS

### 2.1 Search strategy

Smartphone‐based utility applications that are available on *Google Play* and *App Store* were identified in March 2021 using the following expressions and keywords: “Cognitive Behavioral Therapy,” “Obesity,” “Weight,” and “Eating Disorder.” The data were then extracted and analyzed in April and May 2021.

### 2.2 App selection

**Figure 1:**
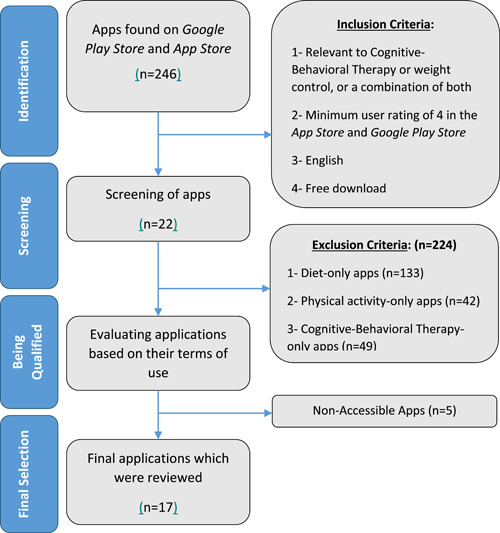
Flowchart indicating the screening process.

The app selection process is described in Figure 1. The inclusion criteria for evaluating the applications in this study included: (1) those being relevant to CBT or weight control, or a combination of both, (2) apps that have a minimum rating of 4 by users, (3) apps that use the English language, and (4) those that are free to download and install. The exclusion criteria in this study included: (1) the apps that were only based on diet, (2) apps that were only based on physical activity, (3) apps that were only based on CBT, and (4) apps that were are not accessible.

### 2.3 Data extraction

All identified apps were registered in an initial list to count the total number of apps and the number of duplicates. The general characteristics of the included apps were extracted from the information in the app stores, while the main app features were verified by the authors by utilizing the app.

### 2.4 MARS app quality assessment

The MARS can be utilized to evaluate the quality of the identified smartphone apps. This scale enables the quality assessment of smartphone health apps with their engagement, functionality, aesthetics, information quality, and subjective features. The MARS scale is a 23‐item tool featuring a 19‐item qualitative part divided into four aspects (engagement, functionality, aesthetics, and information quality) and, finally, one subjective aspect (Table 1). The MARS scale has a reliability of 90 (*α* = 90) and a validity of 79 (interclass correlation coefficient = 79.^12^

**Table 1:** MARS scale evaluation criteria for each aspect.

1. *Engagement* “Entertainment, Interest, Customization, Interactivity, Target Group”
2. *Functionality* “Functionality, Ease of Use, Navigation, Gestural Design”
3. *Aesthetics* “Layout, Graphics, Visual Appeal”
4. *Information quality* “Accuracy of App Description, Goals, Quality of Information, Quantity of Information, Visual Information, Credibility, Evidence‐Based”
5. *Subjective quality* Would you recommend this app to people who might benefit from it? Would you pay for this app? How many times do you think you would use this app in the next 12 months if it were relevant to you? What is your overall star rating for the app?

Abbreviation: MARS, Mobile Application Rating Scale.

### 2.5 Data analysis

According to the MARS scale, all the information is rated based on a 5‐point *Likert* scale (1 = inadequate, 2 = poor, 3 = acceptable, 4 = good, and 5 = excellent). For the quality assessment of the apps, three experts of the research team, including one health information management, one medical informatics, and one software computer engineer answered 23 questions of the MARS scale. Their research areas were e‐health and mobile app development. Additionally, they had at least 2 years of relevant work experience. The higher the score, the better the quality of the smartphone application. Microsoft Excel 2010 was used in this step.

## 3 RESULTS

In the first step, all relevant applications were identified according to the keywords, and 246 apps were screened according to the study inclusion criteria. The identified applications were then screened based on the exclusion criteria. Later, the remaining 22 smartphone applications were downloaded by research members.

Seventeen CBT‐based weight control smartphone apps were examined according to the search strategy and their characteristics (Table 2). All of the identified 17 applications were then rated by research members using the MARS scale and on a 5‐point *Likert* scale (Table 3). Among the apps, two apps named *Noom* and *Peace with Food* were available for free download and installation. However, gaining full access to these apps' features required in‐app payment.

**Table 2:** Description of the apps included in the study.

No.	App name	App platform	Version	Number of downloads	Password protection	Affiliations	Features
1	CBT Companion	Android iOS	3.1.2	+100K	No	Commercial	‐ Record mood ‐ Learn and practice CBT skills ‐ Track progress and get motivated to obtain new skills ‐ Get awards for tasks done ‐ Integrate with the health app (HealthKit) to read your physical activity, nutrition, sleep, and mindfulness minutes and summarize them in the app
2	Rise Up	Android iOS	1.4.0	+100K	Yes	Commercial	‐ Log your meals, emotions, and behaviors ‐ Set custom reminders ‐ Share motivational and inspirational quotes, images, and affirmations ‐ Find support and professional treatment nearby ‐ Export PDF summaries of your Meal Log and Check‐in to share with your treatment team
3	Mindful Eating Coach 2	iOS	1.0.2	+50K	No	Commercial	‐ Before eating, the app helps to decide whether to eat or NOT to eat (resist an urge to eat if you aren't hungry) ‐ Plan to stay mindful while eating ‐ After eating, the app prompts you to remember how hungry you were, why you ate, and how you feel now about what and how much you ate ‐ Monitor your progress ‐ Brief lessons to give more guidance and specific tips
4	Eat Right Now	Android iOS	5.3.0	+50K	No	Commercial	‐ Daily playlists with video and audio ‐ An interactive stress test ‐ Customizable daily goals ‐ Daily night reflection ‐ Bonus exercise tracks ‐ Daily check‐in reminders
5	Recovery Record (RR)	Android iOS	8.9.7	+100K	No	Commercial	‐ Keep a record of meals, thoughts, and feelings ‐ Access attractive reflection images and affirmations ‐ View charts that highlight insights, trends, and progress ‐ Collect jigsaw pieces to earn hidden rewards ‐ Customize your log form, meal plan, reminder schedules, and alarm tones ‐ Share your RR with your treatment team ‐ Receive and send encouraging messages and virtual gifts to/from other users
6	Nourishly	iOS	1.2.9	+100K	No	Commercial	‐ Cognitive‐behavioral meal, activity, sleep, hunger, and temptation tracking ‐ Science‐backed micromissions that tweak your habits to unlock breakthroughs ‐ Simple and specialized meal plans ‐ Thought‐provoking and surprising rewards ‐ Link with your care team to get feedback and precision treatment
7	Eating Disorder Recovery App	iOS	1.0.1	+1K	No	Commercial	‐ Educational videos about eating disorders ‐ Interactive quizzes ‐ Finder tool to connect with healthcare facilities in your area
8	Noom	Android iOS	10.10.0	+10M	No	Commercial	‐ Coaching to help you achieve your personal health goals ‐ 10‐min daily lessons that help you develop healthier habits ‐ A diverse food database with over 1 million scannable barcodes ‐ Health‐tracking tools like weight logging, water tracking, and step counting ‐ Mood logging to better understand your moods and measure progress ‐ Hundreds of healthy, simple recipes that don't require you to restrict your diet
9	Moxie	iOS	1.2.1	+1K	No	Commercial	‐ Integrates with Apple Health and calculates the number of calories you've burned during the day and adds this amount to your junk budget ‐ Understand your emotions ‐ Celebrate and give yourself credit when you stick to a budget or have reached your activity goal ‐ Analyze your behavior
10	Peace with Food	Android iOS	1.3.10	+1K	No	Commercial	‐ Rhythm Tracker guides you to “stay‐in‐the‐gray” throughout the day ‐ Exclusive access to the library of Peace with Food Videos. ‐ Read specially crafted "bite‐sized" Questions & Answers (Q&A) about being at peace with food ‐ Personalized frequency of notifications to check in with your body and "end‐the‐day" settings
11	Savor	Android iOS	1.1.0	+10K	No	Commercial	‐ Hundreds of mindful food journaling sessions ‐ Simple proven mindful food journaling exercises ‐ Share motivational and inspirational quotes and affirmations ‐ Explore the themes of your life that shape your eating patterns
12	Eat Breathe Thrive	iOS	1.0.1	+1K	No	Commercial	‐ A guided mindfulness practice for tracking hunger and fullness signals ‐ A tool (Am I Hungry Quiz) for determining whether you're feeling emotional or physical hunger ‐ An exercise for identifying behaviors that improve health and well‐being ‐ Ability to set reminders to implement healthy behaviors
13	Brighter Bite—ED Recovery	Android iOS	1.1.0	+10K	No	Commercial	‐ Easily track meals (with pictures!) and eating disorder behaviors ‐ Easily track mood and thoughts ‐ Gain insights with eating disorder assessment test ‐ Organize and filter your diary in an easy‐to‐view format ‐ Export tracked data to a shareable PDF report ‐ Gain recovery insights from analysis graphs based on recorded data ‐ Access essential recovery resources, including knowledge, treatment, and communities. ‐ Build up your own resources to cope with distress ‐ See daily motivation quotes to put a smile on your face
14	Mindful	Android iOS	1.2.8	+100K	No	Commercial	‐ All weight‐loss meditation materials ‐ Scientifically designed plan ‐ Mindful eating guide ‐ Lose weight without the time and space limits—you can practice weight‐loss meditations while sleeping, eating, working, and so forth
15	Nutridi	Android iOS	1.2.0	+100K	No	Commercial	‐ Personalized audio content that guides you through each step of your mindful eating journey ‐ Learn practical skills on the go that will help you understand your food cravings and emotional triggers ‐ Find out all you need to know about physical and emotional hunger so you are more in tune with your body's true needs
16	Intuitive Eating	Android	1.0.1	+100	No	Commercial	‐ Daily reminders ‐ Track progress ‐ Log your meals and emotions ‐ Educational videos about eating disorders ‐ Educational lessons
17	Ate	iOS	2.2.3	+100K	No	Commercial	‐ Quickly capture meals using photos ‐ Reflect on and record the reason for eating ‐ Record water, activities, emotions, and daily notes ‐ Create sustainable healthy behaviors for lasting change ‐ Experiment with new habits to see what works best ‐ Look back on previous eating experiments ‐ Automatically track fasting (Intermittent fasting 16/8^a^) ‐ Timeline/profile web sharing (read‐only) ‐ Customize Q&As under meal details for more personalized tracking ‐ Customize experiments and lookback option ‐ Create favorites to reuse for meal photos

Abbreviation: CBT, cognitive‐behavioral therapy.

^a^ Intermittent fasting 16/8 is fasting for 16 h and eating in an 8‐h window.

**Table 3:** Evaluation of smartphone app features based on the MARS scale.

App name	User rating in the store	Engagement	Functionality	Aesthetics	Information quality	Subjective quality	Overall score
CBT Companion	4.8	4.6	4.9	5	4.8	4.7	4.8
Rise Up	4.7	3.4	3.5	2.8	2.4	2	2.8
Mindful Eating Coach 2	4.9	3.6	4.4	3.9	4.6	3.8	4.1
Eat Right Now	4.8	4.5	4.8	4.8	4.9	4.8	4.8
Recovery Record	4.9	4.7	4.3	3.9	3.7	3.9	4.1
Nourishly	4.9	4.7	4.3	4	3.7	3.9	4.1
Eating Disorder Recovery App	4.7	1.2	2.4	2	3.6	2.3	2.3
Noom	4.7	4.5	4.6	4.2	4.3	3.5	4.2
Moxie	5	1.3	2.5	4.1	3.4	2.2	2.7
Peace with Food	4.2	2.5	3	2.3	2.2	1.9	2.4
Savor	5	3.5	3.2	3.4	4	3	3.4
Eat Breathe Thrive	5	2.6	1.7	2.2	2.1	1.8	2.1
Brighter Bite‐ED Recovery	4.7	4.6	4.8	4.2	4.8	4.6	4.6
Mindful	4.6	4.8	5	5	4.9	4.8	4.9
Nutridi	4.9	4.6	4.5	4.6	4.7	4.5	4.6
Intuitive Eating	4.8	2.5	4.1	4	4.3	3.6	3.7
Ate	4.8	4.5	4.7	4.3	4.2	4.2	4.4
Mean score		3.65	3.92	3.80	3.91	**3.5**	3.76

3.1

The evaluation of apps using the MARS scale has been as follows:

### 3.2 Engagement

The scores for this aspect were obtained in five criteria (entertainment, interest, customization, engagement, and target setting) with an average of 3.65. These scores were within the range of 1.2–4.8 out of 5. The *Mindful* application received the highest score in terms of engagement. This smartphone app helps people find their inner motivation, follow their diet consciously, create and maintain a healthy lifestyle, resist food cravings, and overcome laziness and other issues related to losing weight.

### 3.3 Functionality

The scores regarding this aspect of the study were obtained in four criteria with an average of 3.92. The minimum and maximum scores were 1.7 and 5 out of 5. The *Mindful* application received the highest score and is designed to achieve the target weight by following a safe and efficient approach. This smartphone app not only includes weight loss features but also offers scientifically designed plans and informed eating guides.

### 3.4 Aesthetics

The scores for this aspect were obtained in three criteria with an average of 3.80. These scores ranged from 2 to 5. The *CBT Companion* and *Mindful* smartphone apps received the highest scores in aesthetics due to their user interface quality and visual attractiveness.

### 3.5 Information quality

The scores for this aspect were obtained in seven criteria with an average of 3.91. These scores ranged from 2.1 to 4.9 range. The *Eat Right Now* and *Mindful* smartphone apps ultimately received the top scores regarding this aspect of the study. *Eat Right Now* is an educational application that enables users to change their eating habits. Furthermore, this app provides step‐by‐step tutorials which assist the user in controlling their food cravings. The application provides its tutorials through audio and video playlists, target‐setting tools, and daily reminders.

### 3.6 Subjective quality

The scores regarding this aspect were obtained in four criteria (usefulness of the app, frequency of using the application, cost, and user satisfaction) with an average of 3.5. These scores had a minimum and maximum of 1.8 and 4.8, respectively. The *Eat Right Now* and *Mindful* smartphone apps received the top scores regarding this aspect of the study.

## 4 DISCUSSION

This study used the MARS scale to examine the qualitative evaluation of available smartphone CBT‐based applications in the field of overweight control. Relevant applications were downloaded and reviewed according to the inclusion and exclusion criteria of the study. They were then scored according to the MARS scale.

The smartphone apps reviewed in this study focused primarily on sending users daily reminders, recording daily food intake, and registering everyday emotions. Users should be sufficiently aware of the amount of food they consume during the day to be able to control their weight. Thus, users can be guided by sending reminders throughout the day. Additionally, the daily emotions of each person have a profound effect on controlling their weight. Thus, registering such feelings on the app and being provided with exercises to relieve as well as eliminate negative emotions can prevent users from overeating due to such nervous tensions. The results of the current study are in line with previous studies indicating that frequent recording of daily food intake is the key to overweight treatment.^21^ In addition, according to the results of this research, sending motivational messages about the importance of self‐monitoring in addition to incentivizing patients to use the app, may enhance adherence in overweight management.^22^ The results of the reports suggest that daily or weekly feedback and encouragement by sending motivational messages can also promote dietary intake and self‐efficacy.^23^, ^24^

In addition to the mentioned cases, psychological training plays a significant role in controlling people's weight. Users can alter their eating behaviors for the better by using the tutorials provided by these apps. According to the results reported by Wadden et al., combining a weight loss program with a weight loss counseling program can be a powerful combination of tools.^25^

The results revealed all of the apps related to overweight control based on CBT were commercial apps and therefore there is a lack of science‐based apps in the apps market. Thus, there is a need to develop more scientific‐based apps by academic institutions.

One of the smartphone applications that scored the highest on the MARS scale is the *Mindful* app. This application can guide users to eat consciously and provides users with 4‐week exercises which help them relax and focus more on their diet.

In the study, it was identified that reducing the manual entry of diet and physical activity information as well as turning the apps into semiautomatic ones can enhance their engagement, attractiveness, and aesthetics and ultimately increase the chance of them being frequently used by users.

The current study's findings indicated that the Functionality, Information Quality, and Aesthetics of apps should not be considered the only essential aspects when designing such applications. The engagement and Subjective Quality of the apps should also be taken into account. Furthermore, the positive role of behavioral skills in weight control was observed in only 2 out of the 23 applications.

Thus, the findings revealed, as mentioned by Bardus et al.,^26^ the apps with higher quality include the following features: semiautomatic tracking (self‐monitoring), having a forum, and sharing their content on social media (for instance, providing social support), and employing notifications (such as commands/behavioral cues).

Comparing the overall MARS score and the number of downloads indicated that apps with a score of less than 4 generally had less than 50K number of downloads. However, one app titled “Rise Up” had a low overall score of MARS (2.8) and a high number of downloads (+100K).

The use of specialized tools such as the MARS scale to evaluate the quality of smartphone applications is practical and efficient in this regard.^12^, ^27^ Qualitative indicators such as reliability, comprehensiveness, accuracy, and control of data sharing should also be taken into account regarding this matter.

To adequately meet the demands of users, the applications available for download should be user‐friendly, simple, and attractive. The main features that affect the scores of a smartphone app in this category include sending motivational and informative reminders to users. Also, providing psychological training and solutions could help eliminate negative thoughts and establish correct eating behaviors in users. The possibility of recording food intake and emotions daily as well as setting forth approaches would encourage users to use the application again.

### 4.1 Limitations

The most important limitation of this study was that we could not include any apps that did not have a free trial version in the evaluation. Additionally, we may have missed some apps that did not include any of our search criteria related to “CBT” and “Diet” in their titles or descriptions. Furthermore, the app market is constantly changing, with old apps being updated or removed from the app store and new apps being added. As such, the review studies such as this one will need to be regularly updated to keep up with the rapidly changing digital healthcare landscape.

## 5 CONCLUSIONS

Future applications related to this field can be improved by providing a personalization program according to the needs of users and the possibility of online chatting with the therapist. Further improvements can be achieved by ameliorating the areas of engagement, aesthetics, and subjective quality as well as having appropriate privacy policies.

## AUTHOR CONTRIBUTIONS

**Negin Ebrahimi**: Investigation; writing—original draft. **Niloofar Mohammadzadeh**: Conceptualization; methodology; supervision. **Seyed Mohammad Ayyoubzadeh**: Writing—review and editing.

## CONFLICT OF INTEREST STATEMENT

The authors declare no conflict of interest.

## TRANSPARENCY STATEMENT

The lead author Niloofar Mohammadzadeh affirms that this manuscript is an honest, accurate, and transparent account of the study being reported; that no important aspects of the study have been omitted; and that any discrepancies from the study as planned (and, if relevant, registered) have been explained.

## Data Availability

The data that support the findings of this study are available from the corresponding author upon reasonable request.
